# Lactic Acidosis Promotes Mitochondrial Biogenesis in Lung Adenocarcinoma Cells, Supporting Proliferation Under Normoxia or Survival Under Hypoxia

**DOI:** 10.3389/fonc.2019.01053

**Published:** 2019-10-17

**Authors:** Susana Romero-Garcia, Heriberto Prado-Garcia, Alma Delia Valencia-Camargo, Abraham Alvarez-Pulido

**Affiliations:** Department of Chronic-Degenerative Diseases, National Institute of Respiratory Diseases “Ismael Cosío Villegas”, Mexico City, Mexico

**Keywords:** mitochondrial mass, mitochondrial DNA (mtDNA), nuclear respiratory factor (NRF), monocarboxylate transporter (MCT), glutamine, tumor growth rate, glucose deprivation

## Abstract

Lactic acidosis, glucose deprivation and hypoxia are conditions frequently found in solid tumors because, among other reasons, tumors switch to Warburg effect and secrete high levels of lactate, which decreases the pH (<6. 9) in the microenvironment. We hypothesized that lung cancer cells consume lactate and induce mitochondrial biogenesis to support survival and proliferation in lactic acidosis with glucose deprivation even under hypoxia. We examined lung adenocarcinoma cell lines (A-427 and A-549), a breast cancer cell line (MCF-7) and non-transformed fibroblasts (MRC-5). Cells were cultured using RPMI-1640 medium with 28 mM lactate varying pH (6.2 or 7.2) under normoxia (atmospheric O_2_) or hypoxia (2% O_2_). Cellular growth was followed during 96 h, as well as lactate, glutamine and glutamate levels, which were measured using a biochemical analyzer. The expression levels of monocarboxylate transporters (MCT1 and MCT4) were evaluated by flow cytometry. To evaluate mitochondrial biogenesis, mitochondrial mass was analyzed by flow cytometry and epifluorescence microscopy. Also, mitochondrial DNA (mtDNA) was measured by qPCR. Transcript levels of Nuclear Respiratory Factors (NRF-1 and NRF-2) and Transcription Factor A Mitochondrial (TFAM) were determined using RT-qPCR. The specific growth rate of A-549 and A-427 cells increased in lactic acidosis compared with neutral lactosis, either under normoxia or hypoxia, a phenomenon that was not observed in MRC-5 fibroblasts. Under hypoxia, A-427 and MCF-7 cells did not survive in neutral lactosis but survived in lactic acidosis. Under lactic acidosis, A-427 and MCF-7 cells increased MCT1 levels, reduced MCT4 levels and consumed higher lactate amounts, while A-549 cells consumed glutamine and decreased MCT1 and MCT4 levels with respect to neutral lactosis condition. Lactic acidosis, either under normoxia or hypoxia, increased mitochondrial mass and mtDNA levels compared with neutral lactosis in all tumor cells but not in fibroblasts. A-549 and MCF-7 cells increased levels of NRF-1, NRF-2, and TFAM with respect to MRC-5 cells, whereas A-427 cells upregulated these transcripts under lactic acidosis compared with neutral lactosis. Thus, lung adenocarcinoma cells induce mitochondrial biogenesis to support survival and proliferation in lactic acidosis with glucose deprivation.

## Introduction

Lactic acidosis is a common condition found in solid tumors (1–3); for instance, in breast cancer patients, intratumoral lactate levels range from 0.6 to 8.0 μmol/g (4). Lactate can be employed as a carbon source; accordingly, some non-small cell lung cancer (NSCLC) tumors were recently shown to use lactate as a respiratory substrate to survive and proliferate (5). There are different sources that promote lactic acidosis in tumors: first, the altered metabolism of tumor cells augment both glucose consumption and glycolysis with a concomitant increase in lactate production in the presence of oxygen (Warburg effect) (6) or in the absence of oxygen (Pasteur effect) (7, 8). Second, the inefficient formation of microvasculature favors the development of concentration gradients of lactate, glucose, oxygen and pH (9, 10). Third, cancer-associated fibroblasts (CAFs) can also promote high levels of lactate and an acidic microenvironment (pH < 6.9) (6).

Because the tumor microenvironment is variable, cells that initially had access to high glucose levels and normal oxygen concentrations may eventually encounter an environment with lactic acidosis (1, 7), absence of glucose (1) and hypoxia (8). For example, in solid tumors such as colon and stomach cancer, glucose concentrations are much lower within the tumor (0.15 mmol/g) than in normal tissues (1.2 mmol/g) (2). In breast cancer patients, the tumor mass has an estimated median PO_2_ value of 10 mmHg (1.5% O_2_), whereas in normal breast tissue, it is 65 mmHg (8). These changes in the microenvironment might favor tumor survival and invasion; in this regard, hypoxia is known to favor angiogenesis, metastasis, and resistance to radiation and chemotherapies (8).

The acidic microenvironment promotes extracellular matrix degradation and angiogenesis, inhibits the immune response and is toxic for non-transformed cells (7). For instance, human melanoma cells maintained under acidic conditions reduce their capacity to form flank tumors, but they exhibit a greater range of motility and invasive phenotypes (7, 11). Also, lactic acidosis in the presence of glucose (3 mM) promotes autophagy, increases resistance to glucose-deprivation-induced apoptosis and arrests cells in the G0/G1 cell cycle phase as survival mechanisms in murine breast cancer 4T1 cells (10). As a proof of concept, bicarbonate infusions into tumors revert lactic acidosis into lactosis, where tumor cells switch back to the Warburg effect, which induces glucose-deprivation-associated death (3).

Lactate transport requires of monocarboxylate transporters (MCTs). In particular, MCT1 and MCT4 are plasma membrane transporter proteins of lactate and pyruvate. MCT1 is a symporter that co-introduces equimolar lactate and protons. MCT4 may import or export lactate, but it frequently transports lactate out of the cell (5). MCT1 and MCT4 expression are deregulated in several types of cancers. For instance, high MCT4 expression in CAFs accompanied by strong MCT1 expression in tumor cells has been shown to be associated with poor prognosis in prostate cancer (6, 10, 12). A previous report also showed that some NSCLC tissues can incorporate lactate by increasing the expression of MCT1, MCT4, and lactate dehydrogenase A and B (LDHA and LDHB) (5). This study also indicated that other NSCLC tissues neither introduce lactate nor express MCT1, MCT4, LDHA, and LDHB, highlighting the heterogeneity in human NSCLC tumor cells (5). Nevertheless, to our knowledge, regulation of MCTs expression on lung cancer cells by lactate itself has not been studied.

Among the many pathways involved in tumor development, mitochondrial biogenesis has been shown to be important for sustaining cell proliferation. Cells generate more mitochondria from pre-existing organelles in a process called mitochondrial biogenesis to increase their mitochondrial mass to divide them up and inherit sufficient mitochondria to daughter cells (13). Increases in mitochondrial mass and mtDNA are considered markers of mitochondrial biogenesis (14, 15). The main transcriptional regulators of mitochondrial biogenesis include peroxisome proliferator-activated receptor gamma coactivator-1α (PGC-1α), nuclear respiratory factors 1 and 2 (NRF-1 and NRF-2) and mitochondrial transcription factor (TFAM) (14, 16). A significant increase in the PGC-1α, NRF-1 and TFAM proteins with a concomitant increase in mitochondrial biogenesis has been reported in arsenic-induced carcinogenesis of human keratinocytes (14) and endometrial cancer tissue (16). Increases in mtDNA content have been reported in prostatic cancer (12) and in the transformation from hyperplastic to cancer endometrium (15). In contrast, mtDNA depletion has been associated with ovarian cancer progression (17) or with the acquisition of a more invasive phenotype in early prostate carcinoma (18). However, none of these studies describe whether mitochondrial biogenesis is modulated by lactate, acidosis or hypoxia in cancer cells.

Mitochondrial biogenesis appears to be critical for several process of the malignant transformation (19). Mitochondrial biogenesis is increased to favor migratory and invasive tumor phenotypes in breast cancer (8, 20), propagation and survival of stem-like cancer cells (19), and adaptation to hypoxia in a neutral pH microenvironment of human hepatocellular carcinoma (HCC) cells (13). In contrast, a reduction of mitochondrial biogenesis and the subsequent alteration of respiratory capacity has been observed in lung tumors, these features were associated with a lower expression level of Bcl-2 (21). Thus, mitochondrial biogenesis has been thought to be dysfunctional in lung cancer cells.

Since lactic acidosis with extremely limited glucose levels and hypoxia are conditions frequently found in solid tumors, we hypothesized that if lung adenocarcinoma cells survive under glucose-deprivation with lactic acidosis, then they will consume lactate and induce mitochondrial biogenesis independently of the oxygen tension. Thus, we tested *in vitro* the influence of each variable (carbon source, pH and oxygen) on tumor survival and proliferation, we also analyzed the expression of MCT1 and MCT4 and evaluated whether mitochondrial biogenesis is modified in response to lactic acidosis. The results of this study may contribute to develop novel strategies for cancer treatment.

## Materials and Methods

### Cell Lines

Two human lung adenocarcinoma cell lines were used in this study, A-549 and A-427. Additionally, one breast carcinoma cell line (MCF-7) and human fetal lung fibroblast cells (MRC-5) were included. All cell lines were obtained from the American Type Culture Collection (Manassas, VA, USA).

### Growth Kinetics of Tumor Cells

The tumor cell lines and fibroblasts were maintained in RPMI-1640 (Sigma-Aldrich, St. Louis, MO, USA) supplemented with 10% heat-inactivated fetal calf serum (FCS, HyClone, Logan, Utah, USA) with 100 μg/mL of streptomycin and 100 U/mL of penicillin at 37°C, atmospheric O_2_ and 5% CO_2_. The cell lines grew in monolayers and were harvested by trypsinization.

The growth of all carcinoma cell lines and the fibroblasts was tested using RPMI-1640 glucose-free medium (Sigma-Aldrich) supplemented with sodium L-lactate (28 mM) (Sigma-Aldrich), 10% heat-inactivated fetal calf serum, 100 μg/mL of streptomycin and 100 U/mL of penicillin. Because FCS contained a small amount of glucose, the initial glucose concentration was 350 μM. Additionally, RPMI-1640 medium contained L-glutamine and after FCS addition, the initial concentration of L-glutamine was 1.4 mM. The medium was adjusted at pH 7.2 or pH 6.2 using HCl. Normoxic cells were incubated in a humidified chamber at 37°C with filtered atmospheric air (21% O_2_) and 5% CO_2_. Hypoxic cells were incubated in a humidified Billups-Rothenberg chamber (Del Mar, CA, USA) with 2% O_2_, 93% N_2_ and 5% CO_2_ at 37°C.

A-427, A-549 and MCF-7 cells were seeded at a density of 1 × 10^5^ cells/mL, and MRC-5 cells were seeded at a density of 5 × 10^4^ cells/mL. Cellular suspensions prepared in lactate-supplemented medium at pH 7.2 or pH 6.2 were seeded in sextuplicate in a 24-well plate. Two 24-well plates were seeded in an equivalent fashion. One plate was incubated under normoxia, while the other was incubated under hypoxia for 96 h. Depending on the cell line, the supernatant from each well was removed and measured every 8, 12, or 24 h for analysis of metabolites considering evaporation. Cell-free supernatants were stored at −20°C for later analysis. The cells were counted, and cell viability was determined by trypan blue dye exclusion using a TC20 Automated Cell Counter (Bio-Rad Laboratories, Inc., USA). All cultures were repeated at least twice. The specific growth rate was determined during exponential growth according to the following formula:

μ = ln2/(duplication time).

### Determination of Metabolites

The levels of glucose, L-lactate, L-glutamine and glutamate were determined using a YSI 2900 biochemistry analyzer (Yellow Springs Instruments, Ohio, USA) and membranes containing the immobilized enzymes d-glucose oxidase, L-lactate oxidase, L-glutamine oxidase or L-glutamic acid oxidase (YSI, Ohio, USA). For each metabolite, specific standards were prepared and used according to the manufacturer's instructions. The evaporation volume was determined to correct the quantity of each metabolite.

### Analysis of MCT1, MCT4, and CD98 Cell Surface Expression

MCT1 and MCT4 expression was evaluated in cancer and fibroblast cells by flow cytometry, whereas CD98 was evaluated in A-549 and A-427 adenocarcinoma cell lines. Briefly, cells were cultured in 24-well plates under the four above-described conditions over a 48 h time period. The initial cellular concentrations per well were adjusted to 1 × 10^5^ cells/mL for the A-549, A-427, and MCF-7 cell lines and 1.5 × 10^5^ cells/mL for MRC-5. All cultures were repeated at least twice. After incubation, the cells were harvested by non-enzymatic treatment (EDTA-MOPS). The cells were then washed with PBS. Dead cells were excluded by using the Zombie NIR fixable viability kit following manufacturer's instructions (BioLegend, CA, USA). For immunostaining, cells were resuspended with bovine serum albumin (BSA, 1% w/v) and sodium azide (0.1% w/v) and incubated at room temperature for 30 min with rabbit polyclonal antibodies against MCT1 (MCT1-Alexa 647) and MCT4 (MCT4-Alexa 488) from Bioss (Massachusetts, USA), or with monoclonal antibody for CD98 (clone5E5, FITC) from eBioscience (San Diego, CA, USA). After incubation, the cells were washed and fixed with paraformaldehyde (1% w/v) for further cytometric analysis. At least 10,000 events were acquired from the region of viable cells. The median fluorescence intensity (MFI) of MCT1-Alexa 647, MCT4-Alexa 488, and CD98-FITC was determined. The results were analyzed with FlowJo V10 software (TreeStar, Inc., Ashland, Or, USA).

### Mitochondrial Mass Determination

We used MitoTracker Green (Thermo Fisher Scientific Eugene, OR, USA) to determine mitochondrial content in the cells. The initial cellular concentration per well was the same for the different culture conditions and was adjusted to 1 × 10^5^ cells/mL for A-549, A-427, and MCF-7 and 5 × 10^4^ cells/mL for MRC-5. After 48 h of incubation, cells were harvested and washed with phosphate-buffered saline (PBS). After counting, the cells were resuspended in MitoTracker Green 250 nM (200 μL per 2 × 10^5^ cells) and incubated at 37°C for 30 min in the dark. Cells were washed with PBS and resuspended in 200 μL of 7-aminoactinomycin D (7-AAD, BioLegend, CA, USA). Then, stained cells were analyzed by flow cytometry, and at least 2 × 10^4^ events from the viable cellular region (7AAD-negative cells) were acquired in a FACS Canto II Flow Cytometer (Becton Dickinson, San Jose, CA). The median fluorescence intensity (MFI) of MitoTracker Green, which is directly proportional to the mitochondrial content, was determined only in the viable cell population.

### Mitochondrial Mass Using Epifluorescence Microscopy

The cellular preparation for epifluorescence was made as follows. After 48 h of incubation in 48-well plates, the supernatant was removed. Then, each well was washed with 500 μL PBS, and 100 μL of MitoTracker Green 250 nM (Invitrogen, USA) was added. The plates were incubated at 37°C for 30 min. After washing with 500 μL PBS, 100 μL of CellMask Orange 1X (Molecular Probes) was added. The preparations were incubated at 37°C for 5 min. After washing with 500 μL PBS, 100 μL of 0.1 μg/mL Hoechst 33342 solution (Invitrogen) was added. The plates were incubated at 37°C for 10 min; later, digital images were taken using an EVOS FL Imaging System (Life Technologies, USA). Micrographs were analyzed with ImageJ Software v 1.50i (Wayne Rasband, National Institutes of Health, USA) to obtain fluorescence intensity due to MitoTracker Green per cell line.

### DNA and RNA Extraction

One milliliter of 1 × 10^5^ cells/mL for A-549, A-427, and MCF-7 and 5 × 10^4^ cells/mL for MRC-5 was seeded per well in 24-well plates under the four conditions mentioned above. After 48 h of incubation, total DNA and RNA were extracted from cell lines using a ZR-Duet DNA/RNA Miniprep system according to the manufacturer's instructions (Zymo Research, Irvine, CA, USA). Total isolated DNA was stored at −20°C for further analysis. The quality and quantity of RNA were determined by absorbance at 260 and 280 nm using a NanoDrop 2000 (Thermo Scientific, Waltham, MA, USA). The RNA was treated with RNase-free DNase I (Thermo Scientific, Waltham, MA, USA) according to the manufacturer's instructions. The RNA was reverse-transcribed to produce cDNA using the Maxima First Strand cDNA Synthesis Kit for RT-qPCR (Thermo Scientific, Waltham, MA, USA). The cDNA obtained was stored at −20°C until further analysis.

### Quantification of mtDNA Using qPCR (Real-Time PCR)

Mitochondrial mass determination and mtDNA copy number are indicators of mitochondrial content (15). We used qPCR to quantify mtDNA molecules. Briefly, the concentration of purified total DNA was measured using a Nanodrop 2000 Spectrophotometer (Thermo Scientific, USA). The mtDNA (12S) copy number was determined by qPCR and compared to genomic DNA (RNase P), as previously described (22). Briefly, mtDNA was quantified using 125 nM specific primers for the gene 12S (12S-F: 5-CCA CGG GAA ACA GCA GTG ATT-3 and 12S-R: 5-TAT TGA CTT GGG TTA ATC GTG TG-3) and 140 nM of the TaqMan MGB Probe (6'FAM GTG CCA GCC ACC GCG MGENFQ). gDNA copy number was quantified using 1X of the PDARS RNase P Kit (VIC, Thermo Scientific, P/N 4316944) and 1X Universal master mix (Thermo Scientific, USA). mtDNA and gDNA were amplified in a single tube using four 1:5 serial dilutions, beginning with 5 ng/μL of total DNA. All samples, including serial dilutions, were analyzed in triplicate. The amount of mtDNA relative to RNase P was calculated using the following formula: mtDNA/RNase P = 2^−(CtmtDNA−CtRNAseP)^, where Ct is the threshold cycle.

### Transcriptional Analysis of NRF-1, NRF-2, and TFAM Using RT-qPCR

To determine the transcript levels of the main transcriptional regulators of mitochondrial biogenesis, we used semiquantitative RT-qPCR in an ABI Prism 7500 Sequence Detector (Applied Biosystems, Foster City, CA). NRF-1, NRF-2, and TFAM mRNA levels were quantified using specific primers (NRF-1-F: 5′-ATG AAG ACT CGC CTT CTT CTC-3′ and NRF-1-R: 5′-TTG TTG CCT CTT CCG GAT AGA-3′; NRF-2-F: 5′-AGT GCA ATC TGC TAC ACC TAC-3′ and NRF-2-R: 5′-ATG CAG TCT CGA GCG TCC TT-3′; TFAM-F: 5′-TGT GCA CCG GCT GTG GAA GT-3′ and TFAM-R: 5′-TCC CTC CAA CGC TGG GCA AT-3′), SYBR Select Master Mix (Thermo Scientific, Waltham, MA, USA) and cDNA as a template.

The PCR reactions were performed in 96-well reaction plates using the recommended parameters (10 min at 95°C, 40 cycles of 95°C for 15 s and 60°C for 1 min). Validation curves were run using 18S rRNA (18S-F: 5′-TAC CGC AGC TAG GAA TAA TGG-3′ and 18S-R: 5′-CGT CTT CGA ACC TCC GAC TT-3′) and HPRT1 (HPRT1-F: 5′-CCT GCT GGA TTA CAT CAA AGC-3′ and HPRT1-R: 5′-CTG CAT TGT TTT GCC AGT GTC-3′) to determine the suitable endogenous control for all the analyzed genes. The 18S rRNA was selected as the endogenous control for all transcripts. Each PCR reaction was performed in triplicate, and two non-template controls were included. Data were analyzed with Sequence Detection Software v 1.3.1 (Thermo Scientific, Waltham, MA, USA) to establish the PCR cycle at which the fluorescence exceeded a set of cycle thresholds (Ct) for each sample. Target gene expression analysis was performed according to the comparative 2^−ΔΔCt^ method (23).

### Analysis of NRF-1 and NRF-2 Protein Levels

NRF-1 and NRF-2 protein levels were determined in A-549 and A-427 adenocarcinoma cell lines by flow cytometry. After 72 h of incubation under the four tested conditions described above, cells were harvested by trypsinization, washed with PBS, and stained with Zombie NIR to exclude dead cells. Then cells were fixed and permeabilized with Transcription Factor Staining Buffer Set (Invitrogen) according to manufacturer's instructions. After permeabilization, cells were resuspended in 100 μL of rabbit anti-NRF-1 polyclonal antibody (dilution 1:1000, cat. no. bs-1342R, Bioss Antibodies) or rabbit anti-GABPA/NRF2A polyclonal antibody (dilution 1:1000, cat. no. bs-13261R, Bioss Antibodies). After 45 min of incubation, cells were washed and incubated with Alexa 488 mouse anti-rabbit monoclonal antibody (Molecular Probes, Eugene Oregon) for 30 min. Cells were washed with Perm Buffer (provided by the manufacturer) and resuspended in 200 μL PBS/BSA to proceed to the flow cytometry analysis. At least 10,000 events were acquired from the region of viable cells. The MFI values for NRF-1 and NRF-2 were determined. The results were analyzed with FlowJo V10 software.

### Statistical Analysis

All values are expressed as the mean ± standard error of at least two independent experiments. Changes between groups were analyzed using unpaired Student's *T*-test and *post-hoc* tests were performed using GraphPad Prism 7 software. Significant differences between groups were defined at *p* < 0.05.

## Results

### Lactic Acidosis Increased the Proliferation of Lung Adenocarcinoma Cells Compared With Neutral Lactosis

**Figure 1:**
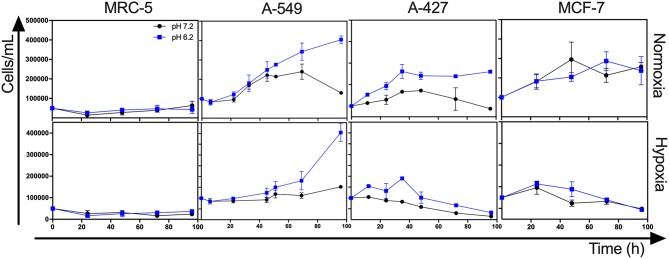
Growth curves of MRC-5, A-549, A-427, and MCF-7 followed for 96 h, using RPMI-1640 low glucose (350 μM) supplemented lactate (28 mM) adjusted at pH 7.2 or pH 6.2 under normoxia (21% O_2_) or hypoxia (2% O_2_).

Lactic acidosis is a condition frequently found in solid tumors. Thus, we wanted to evaluate whether lung adenocarcinoma cells could proliferate in the presence of lactate with or without acidosis under normoxia (21% O_2_) or hypoxia (2% O_2_). We included the non-transformed cells (MRC-5) as a negative control and the breast tumor cell line (MCF-7) as a positive control because these cells can survive consuming lactate (4). We found that the specific growth rate (μ) of A-549 and A-427 cells significantly increased under lactic acidosis compared with neutral lactosis either under normoxia or hypoxia. Although A-549 cells only showed a tendency to increase growth rate under lactic acidosis and normoxia (Table 1), cell number up to 96 h of culture was greater under lactic acidosis (Figure 1). Of note, from the start of the experiment to the beginning of the growth phase, there was an adaptation phase (lag phase) that was variable for each cell line and culture condition (Figure 1). A-427 and MCF-7 tumor cells did not survive in neutral lactosis under hypoxia and they showed a decline in proliferation associated with cell death, because the cell number at 48 h was smaller than time zero (Figure 1). In contrast, after an adaptation phase of 8 h under lactic acidosis and hypoxia, the A-549 tumor cells exhibited a low proliferation rate; nonetheless, this rate increased after 48 h of incubation (Figure 1).

**Table 1:** Specific growth rate of tumor cell lines and fibroblast cells.

	**Normoxia**		**Hypoxia**	
Cell line	pH 7.2 (×10^−2^ h^−1^)	pH 6.2 (×10^−2^ h^−1^)	pH 7.2 (×10^−2^ h^−1^)	pH 6.2 (×10^−2^ h^−1^)
MRC-5	1.9 (0.2)	1.4 (2.2)	[−1.9 (1.1)]^**^	1.4 (1.1)^a^
A-549	1.5 (0.7)	2.0 (0.5)	[0.3 (0.4)]^*^	1.1 (0.6)^a^
A-427	1.2 (0.6)	2.4 (0.4)^*^	[−0.7 (0.4)]^**^	0.8 (1.0)^a^
MCF-7	2.6 (0.3)	1.5 (0.6)	[−0.4 (0.4)]^**^	0.5 (0.8)^*^

*Specific rate of growth was determined on exponential phase. All cultures were made by triplicate in tissue-culture plate using RPMI-1640 supplemented with lactate (28 mM), glucose (0.35 mM), and pH 7.2 or pH 6.2 under normoxia or hypoxia. Values are expressed as mean (std dev)*.

* p < 0.05 and

** *p < 0.01 respect to normoxia pH 7.2 condition*.

a *p < 0.05 respect to hypoxia pH 7.2 condition*.

In the case of the non-transformed MRC-5 cells cultured under lactic acidosis, the μ diminished under normoxia but increased under hypoxia compared with the neutral condition (Table 1, Figure 1). Nonetheless, MRC-5 cells presented a long adaptation phase and entered a survival stage, with the exception of neutral lactosis and hypoxia.

These results indicate that lactic acidosis allows lung adenocarcinoma cells to survive and even proliferate in lactic acidosis and glucose deprivation.

### A-427 and MCF-7 but Not A-549 Cells Cultured Under Normoxia Consumed Lactate Independently of the pH

After finding that adenocarcinoma cells in lactic acidosis proliferated under normoxia or survived longer under hypoxia, we hypothesized that adenocarcinoma cells would consume lactate because all tumor cells and fibroblasts completely consumed the initial small amount of glucose (350 μM) during the first 8 h of incubation. Then, the cultures remained glucose-free to the end of the incubation period of 96 h (data not shown).

**Figure 2:**
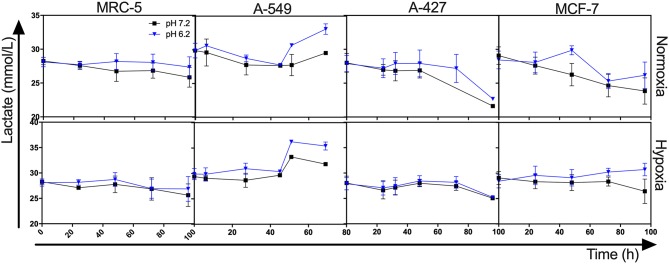
Lactate levels from cultures of MRC-5, A-549, A-427, and MCF-7 followed for 96 h, using RPMI-1640 low glucose (350 μM) supplemented lactate (28 mM) adjusted at pH 7.2 or pH 6.2 under normoxia (21% O_2_) or hypoxia (2% O_2_).

Interestingly, we found that when A-427 and MCF-7 cells were cultured under normoxia independently of pH, they consumed lactate during both the growth and stationary phases (Figures 1, 2). In contrast, A-549 and MRC-5 consumed low lactate quantities when they were cultured under neutral lactosis and normoxia (Figure 2). Surprisingly, none of the cell lines consumed lactate under hypoxia regardless of the pH (Figure 2).

### Under Lactic Acidosis, A-549 Cells Consumed Higher Amounts of Glutamine Than A-427, MCF-7, and MRC-5 Cells

**Figure 3:**
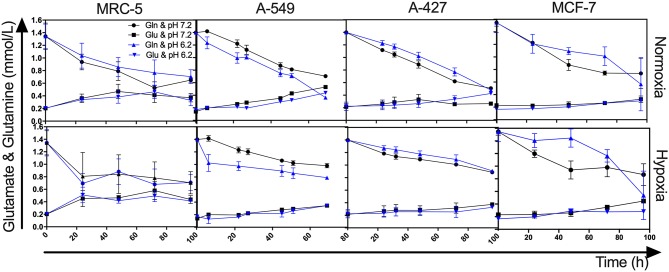
Glutamine and glutamate levels from cultures of MRC-5, A-549, A-427, and MCF-7 followed for 96 h, using RPMI-1640 low glucose (350 μM) supplemented lactate (28 mM) adjusted at pH 7.2 or pH 6.2 under normoxia (21% O_2_) or hypoxia (2% O_2_). Gln, Glutamine; Glu, Glutamate.

Because we observed that under lactic acidosis, A-549 cells proliferated but did not consume lactate, we investigated whether A-549 cells consumed glutamine to support proliferation and survival under lactic acidosis with glucose deprivation. We found that A-549 cells cultured under lactic acidosis consumed more glutamine (1.1 mM at 72 h) and produced more glutamate (0.4 mM at 72 h) than A-427, MCF-7 and MRC-5 cells (0.4–0.6 mM of consumed glutamine and 0–0.1 mM of produced glutamine at 72 h) (Figure 3), indicating that glutaminolysis supports the proliferation of A-549 cells. Additionally, A-549 cells consumed glutamine faster under lactic acidosis than neutral conditions independent of oxygen tension (Figure 3). In contrast, A-427, MCF-7 and MRC-5 cells consumed glutamine faster under neutral lactosis than in lactic acidosis (Figure 3).

### MCT1 Expression Was Differentially Modulated, While CD98 Expression Diminished on Both Adenocarcinoma Cells Under Lactic Acidosis

**Figure 4:**
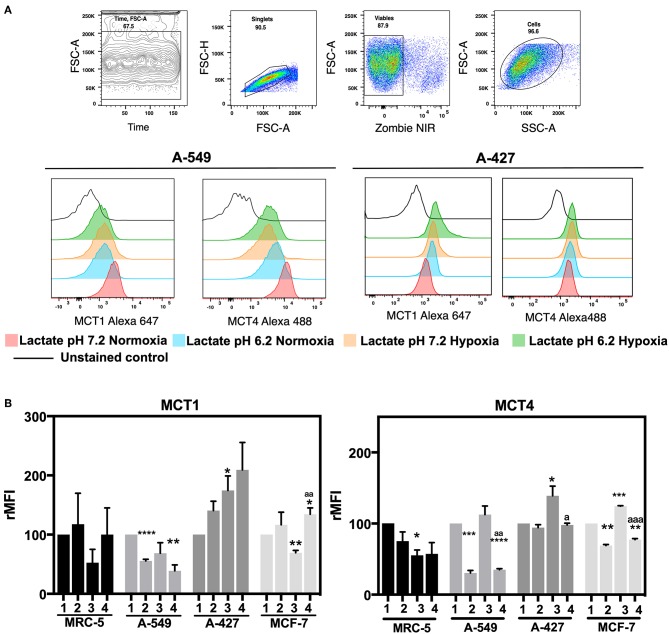
Determination of monocarboxilate transport levels in cancer cell lines and fibroblasts, cultured for 48 h. **(A)** Representative flow cytometric analysis and representative histograms of MCT1 and MCT4 expression on lung adenocarcinoma cell lines. **(B)** Relative median fluorescence intensity (rMFI) values for MCT1 and MCT4 on tumor and fibroblast cells. (1) Normoxia, pH 7.2; (2) Normoxia, pH 6.2; (3) Hypoxia, pH 7.2 and (4) Hypoxia, pH 6.2. Bars represent mean ± SEM of at least three independent experiments. ^*^p < 0.05, ^**^p < 0.01, ^***^p < 0.001, ^****^p < 0.0001 respect to normoxia pH 7.2. ^a^*p* < 0.05, ^aa^*p* < 0.01, ^aaa^*p* < 0.01 respect to hypoxia pH 7.2.

After finding that A-427 and MCF-7 cells consumed lactate under normoxia, we analyzed the expression of MCT1 and MCT4 on the cell surface. MFI values for the expression of MCT1 and MCT4 were normalized with respect to neutral lactosis condition and reported as relative MFI values (rMFI). A representative flow cytometric analysis is shown in Figure 4A.

We found that A-427 and MCF-7 cells tended to increase MCT1 levels under acidosis compared with neutral conditions independent of oxygen tension (Figure 4B). Additionally, A-549 cells significantly diminished MCT1 expression under acidosis regardless of oxygen tension, whereas MRC-5 cells did not change the MCT1 expression levels (Figure 4B). Hypoxia tended to increase MCT4 levels compared with normoxia in all tumor cell lines when they were cultured under neutral pH. Interestingly, A-549, A-427 and MCF-7 cells significantly diminished MCT4 levels under acidosis compared with neutral conditions (Figure 4B).

CD98 alongside with LAT1 form an antiporter that introduces long amino acids at the expense of intracellular glutamine (24). Because CD98 has an important role in glutamine metabolism, we analyzed CD98 protein levels on the surface of A-549 and A-427 adenocarcinoma cells (Table 2). We found that CD98 expression on both A-549 and A-427 adenocarcinoma cells significantly decreased under lactic acidosis independent of oxygen tension (Table 2). These results suggest that lung adenocarcinoma cells inhibit the intracellular glutamine release. Thus, under lactic acidosis and glucose withdrawal, A-427 and MCF-7 cells consumed lactate by increasing MCT1 expression, whereas A-549 cells did not consume lactate neither increased MCT1 expression. However, A-549 cells inhibited glutamine release and increased glutamine consumption which may indicate that glutaminolysis was favored under lactic acidosis.

**Table 2:** rMFI of CD98 protein on the cell surface of lung adenocarcinoma cells.

	**Normoxia**		**Hypoxia**	
Cell line	pH 7.2	pH 6.2	pH 7.2	pH 6.2
A-549	100	61.9 (10.8)^*^	79.0 (2.7)^**^	55.1 (5.3)^**^^a^
A-427	100	35.3 (7.2)^**^	82.8 (12.3)	76.0 (4.5)^*^

*All cultures were made by triplicate in tissue-culture plate using RPMI-1640 supplemented with lactate (28 mM), glucose (0.35 mM), and pH 7.2 or pH 6.2 under normoxia or hypoxia during 48 h. Values are reported as percentages with respect to lactate pH 7.2 and expressed as mean (std dev)*.

* p < 0.05 and

** *p < 0.01 respect to normoxia pH 7.2 condition*.

a *p < 0.05 respect to hypoxia pH 7.2 condition*.

### Tumor Cells Increased Mitochondrial Mass Under Lactic Acidosis

**Figure 5:**
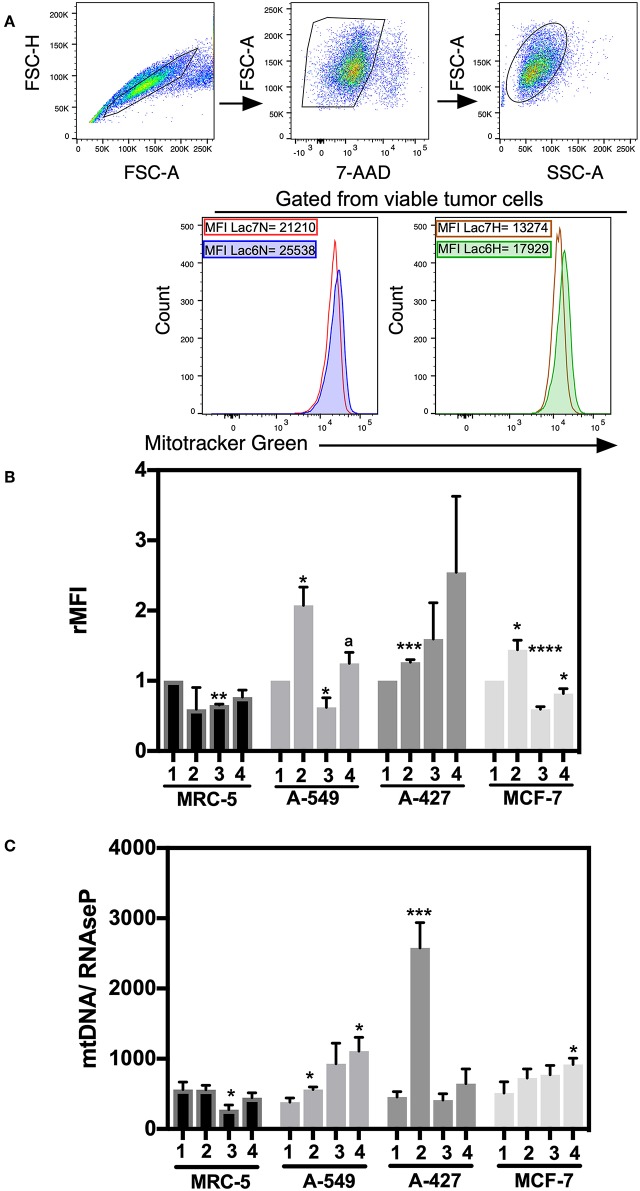
Analysis of mitochondrial mass and mtDNA levels in cancer cell lines and fibroblasts cultured in RPMI-1640 low glucose (350 μM) supplemented with lactate (28 mM) for 48 h. **(A)** Histograms of a representative experiment of the A-427 tumor cell line. Analysis was performed using 7AAD-negative (viable) cells. **(B)** Relative median fluorescence intensity (rMFI) values for MitoTracker Green (MTG) in cancer and fibroblast cells. **(C)** Amount of mtDNA relative to the amount of the nuclear RNase P gene in cancer cell lines and fibroblasts. (1) Normoxia, pH 7.2; (2) Normoxia, pH 6.2; (3) Hypoxia, pH 7.2 and (4) Hypoxia, pH 6.2. Bars represent the mean with SEM of at least three independent experiments. ^*^p < 0.05, ^**^p < 0.01, ^***^p < 0.001, ^****^p < 0.0001 respect to normoxia pH 7.2. ^a^*p* < 0.05 respect to hypoxia pH 7.2.

We next wanted to determine whether mitochondrial biogenesis correlated with tumor growth or tumor survival. Thus, we evaluated mitochondrial mass by staining tumor cell lines and fibroblast cells with MitoTracker Green dye, which is a fluorescent compound that can accumulate in the lipid environment of the mitochondria and emit fluorescence independently of the mitochondrial membrane potential. We included 7-AAD staining to guarantee the analysis of exclusively viable cells. A representative flow cytometric analysis is shown in Figure 5A. MFI values for MitoTracker Green were normalized with respect to neutral lactosis and normoxia condition.

**Figure 6:**
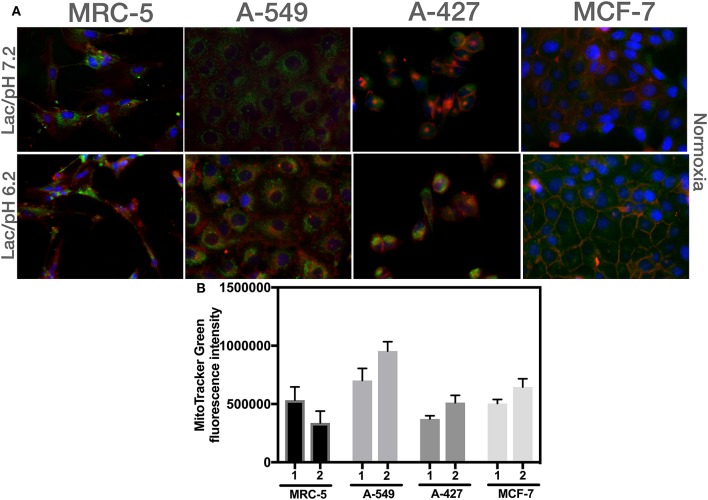
Analysis of mitochondrial mass using epifluorescence microscopy of different cells cultured in RPMI-1640 with lactate and glucose deprivation at (1) pH 7.2 or (2) pH 6.2 under normoxia for 48 h. **(A)** Representative epifluorescence images of A-427, A-549, MCF-7 cells (60X), and MRC-5 fibroblast (40X). **(B)** Analysis of MitoTracker Green fluorescence intensity per cell line.

We found that A-549, A-427, and MCF-7 cell lines cultured under lactic acidosis significantly increased mitochondrial mass compared with neutral lactosis condition, either under normoxia or hypoxia; by contrast, MRC-5 fibroblasts did not increase their mitochondrial mass under lactic acidosis (Figure 5B). We corroborated these data using epifluorescence microscopy. Representative epifluorescence images of A-427, A-549, MCF-7 cells and MRC-5 fibroblasts cultured under normoxia are shown in Figure 6A, MitoTracker Green fluorescence intensity tended to increase in tumor cells but not in fibroblasts when cells were cultured in lactic acidosis with glucose deprivation under normoxia (Figure 6B).

These data indicated that mitochondrial mass content increased when tumor cells proliferated under normoxia or presented a survival stage under hypoxia when cells were cultured in lactic acidosis.

### Adenocarcinoma Cells Increased mtDNA Levels Under Lactic Acidosis

To corroborate the mitochondrial mass findings made by flow cytometry and epifluorescence microscopy, we also measured the mtDNA levels in the tumor cell lines and fibroblast cells by qPCR and used the RNase P gene for normalization.

Under lactic acidosis, A-549 and A-427 cells significantly increased mtDNA levels compared with neutral lactosis under normoxia (Figure 5C). When these adenocarcinoma cells were cultured under lactic acidosis and hypoxia, mtDNA levels tended to increase compared with neutral lactosis (Figure 5C). These findings correlated with the findings of increased mitochondrial mass of adenocarcinoma cells cultured in lactic acidosis under normoxia and hypoxia. In contrast, the mtDNA levels of MCR-5 cells did not increase when these cells were cultured under lactic acidosis, either under normoxia or hypoxia (Figure 5C). In the case of the MCF-7 cell line, the mtDNA levels showed a tendency to increase under lactic acidosis.

### A-549 and MCF-7 Cell Lines Overexpressed NRF-1 NRF-2 and TFAM Compared With MRC-5

**Figure 7:**
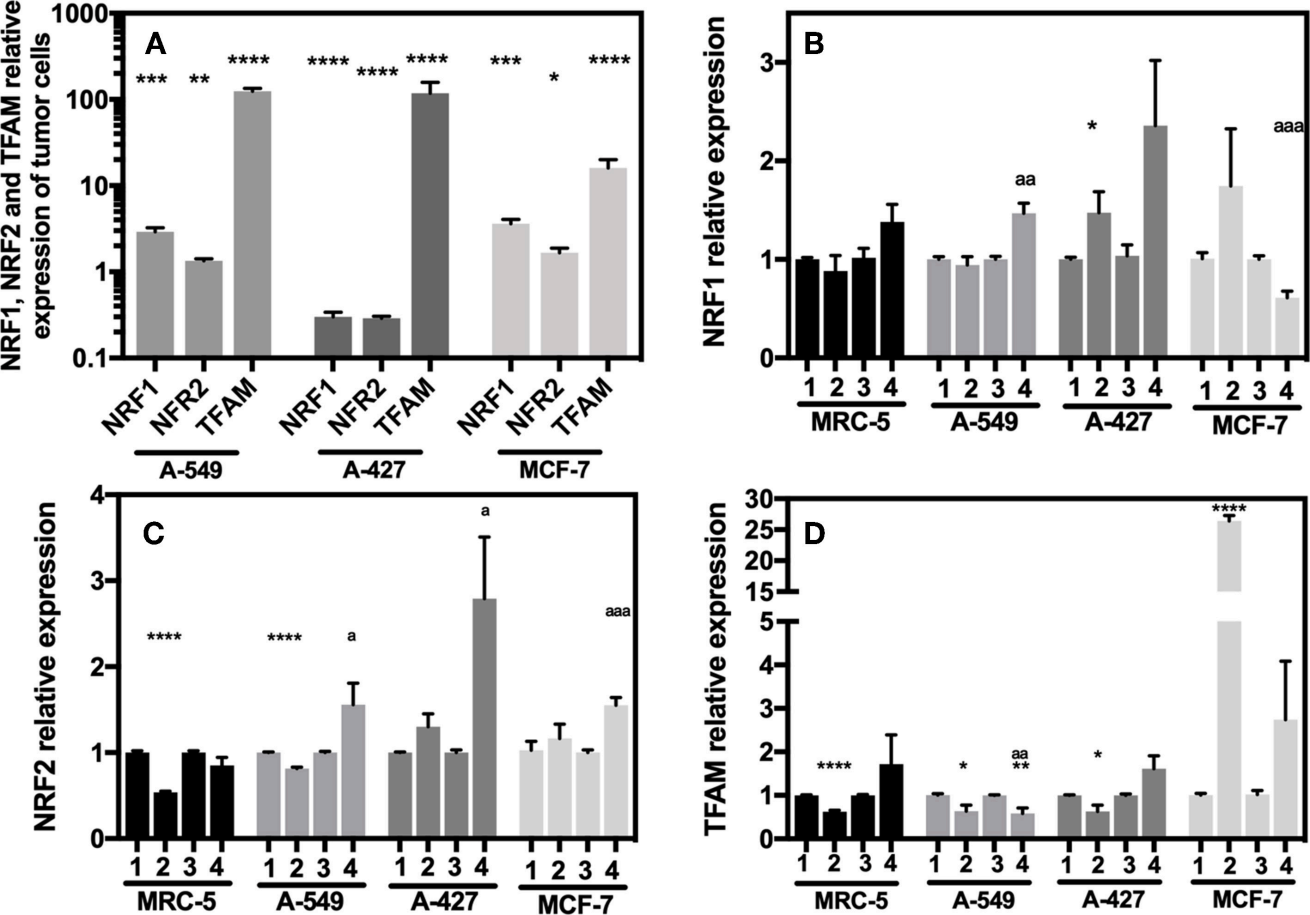
Relative quantification of NRF-1, NRF-2, TFAM in cancer cell lines and fibroblasts cultured for 48 h. **(A)** Relative expression of NRF-1, NRF-2, TFAM in tumor cells compared with their expression in MRC-5, all cultured under neutral lactosis and normoxia. Relative expression of NRF-1 **(B)**, NRF-2 **(C)**, and TFAM **(D)** in the tumor cells and fibroblast cultured under lactic acidosis with respect to same cells cultured in neutral lactosis under normoxia or hipoxia. (1) Normoxia, pH 7.2; (2) Normoxia, pH 6.2; (3) Hypoxia, pH 7.2 and (4) Hypoxia, pH 6.2. Bars represent transcriptional data of two independent culture experiments expressed in mean ± SEM. ^*^p < 0.05, ^**^p < 0.01, ^***^p < 0.001, ^****^p < 0.0001 respect to the transcript levels in MRC-5 or normoxia pH7.2. ^a^*p* < 0.05, ^aa^*p* < 0.01, ^aaa^*p* < 0.01 respect to hypoxia pH7.2.

We next evaluated the transcript levels of the main biogenesis regulators, such as nuclear respiratory factor (NRF) 1 and 2 and mitochondrial transcription factor A (TFAM). After normalization using 18S rRNA, we determined the relative expression of the abovementioned genes in the tumor cells compared with their counterparts expressed in MRC-5 fibroblasts when all cells were cultured under neutral lactosis and normoxia. We found that the NRF-1 and NRF-2 transcript levels were significantly upregulated in A-549 and MCF-7 cell lines compared with their counterparts in MRC-5 fibroblasts (Figure 7A). Remarkably, all tumor cell lines significantly increased TFAM transcript levels compared with MRC-5 cells (Figure 7A), possibly to protect and stabilize mtDNA molecules and consequently avoid their degradation. Previous reports showed that this is an mtDNA replication-independent pathway that increases mitochondrial biogenesis, enhancing proliferation (14, 25).

Next, we evaluated how the expression of the different genes in tumor and fibroblast cells varied in acidosis compared with neutral conditions. We found that A-427 cells significantly increased NRF-1 and NRF-2 mRNA levels in lactic acidosis under normoxia and hypoxia (Figures 7B,C). At protein level, only NRF-2 intracellular protein significantly increased in A-427 cell line cultured under lactic acidosis with glucose deprivation independent of oxygen tension (Table 3). MCF-7 cells significantly increased TFAM transcript levels in lactic acidosis under normoxia or hypoxia (Figure 7D). In contrast, MRC-5 cells cultured under lactic acidosis did not increase the NRF-1, NRF-2, and TFAM transcript levels.

**Table 3:** rMFI of NRF-1 and NRF-2 intracellular protein levels in A-427 adenocarcinoma cell line.

	**Normoxia**		**Hypoxia**	
Protein	pH 7.2	pH 6.2	pH 7.2	pH 6.2
NRF-1	1.0	0.97 (0.09)	1.0	1.05 (0.03)
NRF-2	1.0	1.3 (0.04)^*^	1.0	1.23 (0.04)^*^^a^

*All cultures were made by triplicate in tissue-culture plate using RPMI-1640 supplemented with lactate (28 mM), glucose (0.35 mM), and pH 7.2 or pH 6.2 under normoxia or hypoxia during 72 h. Values are expressed as mean (std dev)*.

* *p < 0.05 respect to normoxia pH 7.2 condition*.

a *p < 0.05 respect to hypoxia pH 7.2 condition*.

These findings indicate that tumor cells differentially express the NRF-1, NRF-2, and TFAM genes. Although some tumor cell lines (A-549 and MCF-7) maintained increased levels of NRF-1, NRF-2 and TFAM, lactic acidosis promoted the increase in NRF-1, NRF-2, and TFAM transcript levels, only when the tumor cells expressed low levels of the transcription factors. This was the case for the A-427 cell line, where intracellular NRF-2 protein levels, but not NRF-1, correlated with increased mRNA levels.

## Discussion

Lactic acidosis with very low glucose quantities is a condition found in solid tumors (2). Some reports have shown the importance of lactic acidosis in the transformation to a more aggressive tumor phenotype, favoring invasion and metastasis (7, 9). Although some groups have tried to find the mechanisms by which tumor cells proliferate in media containing lactic acidosis with high glucose levels from 3 to 10 mM (1, 3, 5); there are no reported data concerning the means by which tumor cells survive and proliferate under lactic acidosis with extremely low concentrations of glucose (350 μM), under normoxia (O_2_, 21%) and hypoxia (O_2_, 2%), though these are common conditions found in solid tumors. Lactic acidosis with an appreciable supply of glucose (3 mM) promotes a significantly longer sustainable proliferation of a murine breast cell line (4T1) than cells cultured only with glucose, suggesting that lactic acidosis but not acidosis alone favors cellular survival (3, 10). Accordingly, we found that lung adenocarcinoma cell lines (A-549 and A-427) cultured in lactic acidosis with extremely low glucose concentrations (350 μM) showed an increased growth rate under normoxia or survived for a longer period under hypoxia than cells cultured under glucose deprivation with neutral lactosis. In particular, lactic acidosis was especially beneficial for MCF-7 cells when they were cultured under hypoxia. In contrast, MRC-5 non-transformed cells cultured under lactic acidosis did not survive. Furthermore, each separate variable, lactosis, or acidosis alone, does not support tumor survival under glucose deprivation. These data indicated that lactic acidosis under normoxia or hypoxia, but not lactosis alone, activates an adaptive response that supports tumor survival, which is not present in normal cells. Although this study has the limitation of being *in vitro*, our results may help to explain why the experimental treatment of tumors with bicarbonate infusion has presented some benefits (3, 7). Taken together, our results indicate that lactic acidosis is a potent survival factor that allows lung adenocarcinoma and breast tumor cells to develop resistance to glucose deprivation even under hypoxia. It will be important to extend this study in animal models and to correlate our results with human tumor samples.

MCT1 expression has been associated with increased mitochondrial OXPHOS, whereas MCT4 is abundantly expressed on glycolytic cells to allow lactate expulsion and is also upregulated by hypoxia (12, 26, 27). This last phenomenon was also observed in our data. Accordingly, we found that A-427 and MCF-7 cells cultured under lactic acidosis and normoxia consumed lactate, which was associated to an increase in MCT1 expression and diminished MCT4 expression. Thus, our results suggest that A-427 and MCF-7 cells have an oxidative phenotype that allows proliferation and survival. Interestingly, A-549 cells did not consume lactate and diminished MCT1 and MCT4 expression levels. These results complement the findings reported by Faubert et al., who showed that some biopsies from NSCLC can use lactate by increasing the expression of MCT1, MCT4, LDHA, and LDHB, but due to the heterogeneity in human NSCLC cells, there are NSCLC tumors that neither introduce lactate nor express MCT1, MCT4, LDHA, and LDHB (5). Nevertheless, we found that A-549 lung adenocarcinoma cells did not introduce lactate nor upregulated MCT1 or MCT4; instead these cells consumed glutamine under lactic acidosis and glucose deprivation, allowing proliferation under normoxia or hypoxia. We corroborated the important role of glutamine in tumor survival under lactic acidosis with glucose deprivation, analyzing CD98 expression, which has an important participation in glutamine release. CD98 (4F2hc), covalently associated to glutamine transporter LAT1 (SLC7A5), contributes to glutamine efflux and large neutral amino acids influx, whereas ASCT2 (SLC1A5) allows Na^+^-glutamine influx and neutral amino acids efflux (24, 28). Although increased LAT1 and ASCT2 expressions have also been reported in human melanoma samples, prostate cancer and breast cancer (29–31), the role of glutamine in cellular homeostasis is complex. We found that under lactic acidosis and glucose deprivation, lung adenocarcinoma cells inhibited glutamine release by diminishing CD98 expression, alongside with the increased intake of glutamine, both results indicate that lung adenocarcinoma cells increase glutaminolysis under lactic acidosis. Nevertheless, it will be important to evaluate ASCT2 expression on lung cancer cells cultured under lactic acidosis. Hence, our study shows that intracellular glutamine supports tumor survival and proliferation under lactic acidosis with glucose deprivation.

Mitochondrial biogenesis has been evaluated through different parameters, such as mitochondria and mtDNA count (13–15), mitochondrial mass determination (13), and the analysis of transcript or protein levels for NRF-1, NRF-2, TFAM, PGC-1α, and CS (13, 14, 16). Here, we found that lactic acidosis induced mitochondrial biogenesis in tumor cell lines (A-549, A-427, MCF-7), as evidenced by an increase in both mitochondrial mass and mtDNA, accompanied by high transcript levels for NRF-1, NRF-2, and TFAM in A-549 and MCF-7 cells or upregulation of these transcripts by lactic acidosis in A-427 cells. Tumor cells stimulate mitochondrial biogenesis not only for proliferation but also for promoting malignant transformation (15), in migration and invasiveness (8, 20) and during tumor adaptation to hypoxia (13). Here, we showed that lung adenocarcinoma cells (A-549, A-427) and breast cancer cells (MCF-7) stimulate mitochondrial biogenesis to survive under hypoxia; thus, lung and breast cancer cells share this survival response with other very aggressive types of cancer, such as HCC (13). Of note, mitochondrial biogenesis of fibroblast cells (MRC-5) was not increased under lactic acidosis; consequently, these cells were not able to proliferate under normoxia or survive under hypoxia. On the other hand, MCF-7 cells cultured with lactic acidosis in the presence of glucose increase their mitochondrial mass compared with medium with glucose alone (6). Our results complement these findings because we found that, even under glucose deprivation and hypoxia, lactic acidosis increased mitochondrial biogenesis in MCF-7 cells.

Bellance et al. suggested that mitochondrial biogenesis was diminished or damaged in lung cancer because they found diminished levels of mRNA for PGC-1α, reduced expression of PGC-1α and TFAM proteins, as well as, lower quantities of total mitochondrial area/cell area in lung cancer biopsies with respect to non-cancer tissue (21). Conversely, our study proposes that lung cancer cells need to face stressful conditions, such as lactic acidosis with glucose deprivation, to induce mitochondrial biogenesis.

## Conclusions

Cancer cells need to survive when both glucose level and oxygen concentration are low. Under these last conditions, lactic acidosis becomes a key factor in tumor survival promoting mitochondrial biogenesis, although some tumor cells prefer to consume other alternative carbon sources, such as glutamine, rather than lactate. In contrast, non-transformed cells such as MRC-5 failed to induce mitochondrial biogenesis under these stressful and common tumor conditions. Thus, we report a tumor behavior that supports tumor survival.

Understanding metabolic adaptive mechanisms for recovering lung tumor cell proliferation under conditions that mimic the tumor microenvironment may provide promising opportunities to improve traditional cancer therapies or find new therapeutic targets to develop specific treatments.

## Data Availability Statement

The datasets generated for this study are available on request to the corresponding author.

## Author Contributions

SR-G designed the study. SR-G, HP-G, AV-C, and AA-P performed the experiments. SR-G and HP-G wrote and critically reviewed the manuscript. All authors contributed to manuscript revision, read, and approved the submitted version.

### Conflict of Interest

The authors declare that the research was conducted in the absence of any commercial or financial relationships that could be construed as a potential conflict of interest.
